# Valproic Acid Induces Autism-Like Synaptic and Behavioral Deficits by Disrupting Histone Acetylation of Prefrontal Cortex ALDH1A1 in Rats

**DOI:** 10.3389/fnins.2021.641284

**Published:** 2021-04-28

**Authors:** Huan Liu, Mei Tan, Boli Cheng, Si Wang, Lu Xiao, Jiang Zhu, Qionghui Wu, Xi Lai, Qian Zhang, Jie Chen, Tingyu Li

**Affiliations:** ^1^Children’s Nutrition Research Center, Children’s Hospital of Chongqing Medical University, Chongqing, China; ^2^Chongqing Key Laboratory of Child Nutrition and Health, Ministry of Education Key Laboratory of Child Development and Disorders, National Clinical Research Center for Child Health and Disorder, Chongqing, China

**Keywords:** histone acetylation, ALDH1A1, retinoic acid, RARα, homeostatic synaptic plasticity, autism spectrum disorder, valproic acid

## Abstract

**Objectives:**

This study aimed to investigate the impact of valproic acid (VPA) on the histone acetylation of acetaldehyde dehydrogenase 1A1 (ALDH1A1) and the mechanism underlying VPA-induced autism-like behavior.

**Methods:**

Female Sprague-Dawley rats were intraperitoneally injected with VPA during gestation to establish an autism model in their offspring. Some offspring prenatally exposed to VPA were randomly treated with MS-275, one histone deacetylase (HDAC) inhibitor, or retinoic acid (RA) after birth. Behavioral tests were conducted on the offspring 6 weeks after birth. Electrophysiological experiments were performed to investigate long-term potentiation (LTP) in the prefrontal cortex (PFC). The expression levels of AMPA receptors (GluA1 and 2), NMDA receptors (GluN1 and 2), synapsin 1 (SYN1), HDAC, acetylated histone 3 (AcH3), RA receptor alpha (RARα), and ALDH1A1 in the PFC were measured by Western blotting and quantitative polymerase chain reaction. ALDH enzyme activity in PFC tissue was detected using a Micro ALDH Assay Kit. The RA level in the PFC was measured using ultrahigh-performance liquid chromatography/tandem mass spectrometry. A chromatin immunoprecipitation (ChIP) experiment explored the interaction between the ALDH1A1 gene and AcH3.

**Results:**

Offspring prenatally exposed to VPA showed autism-like behavior, upregulated the levels of LTP and GluN2A, GluA1, and SYN1 proteins relevant to synaptic plasticity in the PFC. The expression levels of HDAC3 mRNA and protein were increased. On the other hand, there was a significant reduction in the levels of AcH3, RARα, RA, ALDH1A1 mRNA and protein, the level of ALDH activity and AcH3 enrichment in the ALDH1A1 promoter region in VPA-induced offspring. Administration of MS-275 in VPA offspring significantly elevated the levels of AcH3, ALDH1A1 mRNA and protein, ALDH activity, RA, the level of RARα protein and the binding of AcH3 to the ALDH1A1 promoter. In addition, the GluA1 protein level and LTP were reduced, and most behavioral deficits were reversed. After RA supplementation in the VPA-treated offspring, the RA and RARα protein levels were significantly upregulated, GluA1 protein and LTP were downregulated, and most autism-like behavioral deficits were effectively reversed.

**Conclusion:**

These findings suggest that VPA impairs histoneacetylation of ALDH1A1 and downregulates the RA-RARα pathway. Such epigenetic modification of ALDH1A1 by VPA leads to autism-like synaptic and behavioral deficits.

## Introduction

Autism spectrum disorder (ASD) is a category of neurodevelopmental disorders that are characterized by social and communication impairments and restricted or repetitive behaviors (Sorrell, 2013; Lai et al., 2014). The pathogenesis of ASD is complex, highly heterogeneous, and still unclear (Nicolini and Fahnestock, 2018; Rylaarsdam and Guemez-Gamboa, 2019). Epigenetic abnormalities may be involved in the pathogenesis of multiple ASD subtypes via dysregulation of widespread genes (Ma et al., 2018; Rylaarsdam and Guemez-Gamboa, 2019). Histone deacetylases (HDACs) perform a major epigenetic process by deacetylating histones (Ma et al., 2018) and play pivotal roles in neurodegenerative diseases, including ASD (Guan et al., 2009; Fischer et al., 2010; Gräff et al., 2012; Autism Spectrum Disorders Working Group of The Psychiatric Genomics Consortium, 2017).

Valproic acid (VPA) is a first-line drug for treating refractory epilepsy (Chanda et al., 2019), and it is also a classic HDAC inhibitor (Chanda et al., 2019). Prenatal exposure to VPA increases the risk of cognitive defects and ASD (Rasalam et al., 2005; Koren et al., 2006; Smith and Brown, 2014). The VPA-induced autism animal model may more effectively reflect the heterogeneity of ASD than the autism models caused by single-gene mutations (Nicolini and Fahnestock, 2018). This model has been widely used to explore the neurobiology underlying ASD (Chanda et al., 2019). *In vitro* studies confirmed that VPA causes neurodevelopmental defects primarily via the inhibition of class I HDACs (Chanda et al., 2019). This effect suggests that HDAC activity and the level of histone acetylation may be dysregulated in the VPA-induced autism model. However, the precise mechanism of histone acetylation dysregulation leading to autism-like behaviors is not clear.

Yoshino et al. (2020) reported that the histone acetylation level regulates the expression level of acetaldehyde dehydrogenase 1A1 (ALDH1A1) in the cholangiocarcinoma (Schilderink et al., 2016). ALDH1A1 is a key rate-limiting enzyme that oxidizes retinaldehyde to retinoic acid (RA) (Kumar and Duester, 2011). Studies have shown that RA directly activates the translation of the AMPA receptor subunit GluA1 by binding to extranuclear RA receptors (RARα) and thus regulates homeostatic synaptic plasticity (HSP) (Maden and Holder, 1991; Clagett-Dame and DeLuca, 2002; Luo et al., 2004; Aoto et al., 2008; Chen and Napoli, 2008; Siegenthaler et al., 2009; Gutierrez-Mazariegos et al., 2011; Chen et al., 2014). HSP dysregulation caused by synaptic structure and function abnormalities is a significant hallmark of ASD (Bagni and Zukin, 2019). Therefore, we proposed that VPA-induced histone acetylation disorder leads to autism-like synaptic and behavioral deficits via regulation of ALDH1A1-RA-RARα signaling.

To investigate this hypothesis, we established a VPA-induced autism model in rats. We assessed the changes in HSP, acetylated histone 3 (AcH3) and ALDH1A1-RA-RARα signaling in the prefrontal cortex (PFC) of VPA-exposed offspring. Then, we used the HDAC inhibitor MS-275 (Simonini et al., 2006) to increase AcH3 levels, induce ALDH1A1-RA-RARα signaling and rescue autism-like synaptic and behavioral deficits in VPA-exposed offspring. Finally, RA supplementation was used to induce RARα signaling and improve autism-like synaptic and behavioral deficits in VPA-exposed offspring. The present study first revealed epigenetic modification of ALDH1A1 as a critical mechanism underlying the VPA-induced ASD subtype.

## Materials and Methods

### Animals and Drug Treatment

Sprague-Dawley (SD) rats were obtained from the Animal Care Center of Chongqing Medical University (Chongqing, China) and housed with ad libitum access to food on a 12/12-h light/dark cycle (light: 7 a.m.–7 p.m.; dark: 7 p.m.–7 a.m.). All experiments were approved by the Institutional Animal Care and Use Committee of Chongqing Medical University.

**FIGURE 1:**
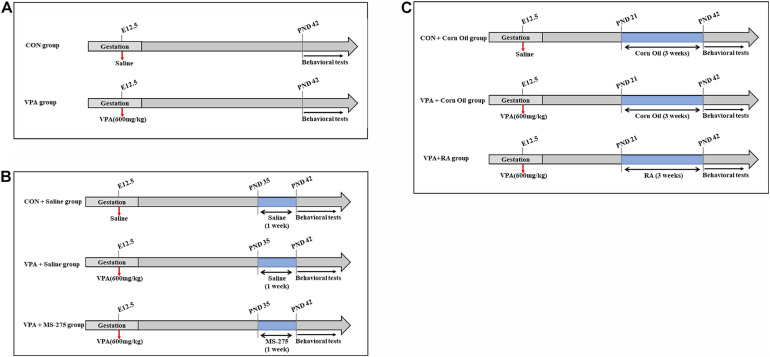
Schematic diagram of experimental design. **(A)** Schematic diagram of the CON and VPA groups. VPA rats (offspring, *n* = 28–30) received a single dose of 600 mg/kg VPA by intraperitoneal injection at 12.5 days of gestation, and the CON rats (offspring, *n* = 28–30) received the same volume of 0.9% saline. **(B)** Schematic diagram of the CON + Saline, VPA + Saline, and VPA + MS-275 groups. VPA + MS-275 offspring (*n* = 28–30) received VPA during pregnancy and an intraperitoneal injection of 3.5 mg/kg MS-275 for a week before each test. Offspring (*n* = 28–30 per group) from the CON + Saline group and VPA + Saline group were treated with saline for comparison. **(C)** Schematic diagram of the CON + Corn Oil, VPA + Corn Oil, and VPA + RA groups. VPA + RA offspring (*n* = 28–30) received VPA during pregnancy and oral administration of 6 mg/kg RA once daily for 3 weeks before each test. Offspring (*n* = 28–30 per group) from the CON + Corn Oil group and VPA + Corn Oil group were treated with corn oil for comparison. Behavioral tests were conducted beginning at PND 43, and the offspring was sacrificed for brain tissue collection after behavioral tests. VPA, valproic acid; RA, retinoic acid; PND, postnatal day.

Female adult rats were mated with males. Pregnant rats were randomly assigned to two groups according to random digits: the VPA (*n* = 9–10) group and the control (CON, *n* = 9–10) group (Figure 1A). The pregnant rats in the VPA group were intraperitoneally injected with a single dose of 600 mg/kg VPA (Sigma, diluted to 250 mg/mL with 0.9% saline) at 12.5 days of gestation, and the rats in the CON group were injected with the same volume of 0.9% saline at the same gestational timepoint.

In the MS-275 treatment experiment, female rats were paired with males and randomly assigned to three groups (*n* = 9–10 per group): the CON + Saline group, VPA + Saline group, and VPA + MS-275 group (Figure 1B). The rats in the VPA + Saline group and VPA + MS-275 group were intraperitoneally injected with VPA during pregnancy, and those in the CON + Saline group were injected with saline. Offspring (*n* = 28–30) from the VPA + MS-275 group received an intraperitoneal injection of 3.5 mg/kg MS-275 after birth [AbMole, dissolved in DMSO to make a stock solution (DMSO concentration of the working solution: < 0.2%) and diluted to 1.5 mg/mL with saline] once daily for 1 week before each test. Offspring (*n* = 28–30 per group) from the CON + Saline group and VPA + Saline group were treated with saline for comparison.

In the RA administration experiment, pregnant female rats were randomly assigned to three groups (*n* = 9–10 per group): the CON + Corn Oil group, VPA + Corn Oil group and VPA + RA group (Figure 1C). The rats in the VPA + Corn Oil and VPA + RA groups were intraperitoneally injected with VPA during pregnancy, and those in the CON + Corn Oil group were treated with saline. Offspring (*n* = 28–30) from the VPA + RA group received an oral administration of 6 mg/kg RA after birth (Sigma, dissolved in corn oil at 2.5 mg/mL) once daily for 3 weeks before each test. Offspring (*n* = 28–30 per group) from the CON + Corn Oil group and VPA + Corn Oil group were treated with corn oil for comparison.

The drug doses and treatment time used were based on previous studies (Ma et al., 2018; Xu et al., 2018; Cheng et al., 2020) and in-house range-finding experiments (see Supplementary Figures 1–5). The offspring used for all experiments in this study were males (Kataoka et al., 2013; Beggiato et al., 2017).

### Behavioral Tests

Autism spectrum disorder is characterized by a triad of core defects in social communication, reduced social interactions and stereotyped or repetitive behaviors (Lai et al., 2018; Xu et al., 2018; Cheng et al., 2020). Behavioral tests in animal studies are often needed to assess these autism-like symptoms. The open-field test is used to evaluate repetitive behaviors and the ability to explore novel environments. The three-chamber social test is used to assess social interaction and social novelty recognition (Deacon, 2006; Xu et al., 2018). Therefore, these two behavioral tests were used in this study, as in most other studies (Lai et al., 2018; Xu et al., 2018; Cheng et al., 2020).

The present study performed behavioral tests with offspring beginning at 6 weeks of age. The open-field test was performed to assess general, exploratory and repetitive behaviors (Deacon, 2006). The total time spent on self-grooming, the time spent in the central zone (the middle square of the 9-square grid), and the total distance traveled during the 5-min test were recorded for each rat. The three-chamber social test was performed to assess social deficits (Deacon, 2006). The apparatus contained three chambers with doorways that allowed access to the side chambers. The test included two phases that lasted 5 min. On the day before the test, the rats were acclimated in the apparatus for 5 min, with an empty cage in each of the two side chambers. The first phase featured an age- and sex-matched stranger rat and an object in the two side chambers, and the second phase contained a stranger rat and a familiar rat in the two side chambers. The time spent in the different chambers was recorded for each test. The apparatus was cleaned after each test. All data were recorded automatically using the ANY-Maze Video Tracking System (Stoelting Co., United States) and analyzed by an experimenter who was blinded to the group assignments.

### Animal Tissue Collection

Offspring rats aged approximately 8 weeks were anesthetized and decapitated. PFC tissue was retained. All brain tissues were stored immediately at −80°C.

### RNA Extraction and Quantitative Polymerase Chain Reaction (qPCR)

Total RNA was isolated and purified from PFC tissue using an RNA extraction kit (Promega Biotech, China). The Prime Script RT Reagent Kit (Takara, Shiga, Japan) was used to generate cDNA from the tissue mRNA. Real-time qPCR was performed to compare mRNA levels using the SYBR-Green Real-time PCR Kit (Takara) and a CFX96 real-time PCR detection system (Bio-Rad, United States). GAPDH was used as the housekeeping gene to quantitatively analyze the target gene expression, and the mRNA expression level was calculated using the 2^–Δ(Δ*CT*)^ method (Ma et al., 2018). Primer sequences for all of the genes profiled in this study were designed using Primer Premier 5 software and are listed below:

GAPDH, sense 5′–CCTGGAGAAACCTGCCAAG–3′ and antisense 5′–CACAGGAGACAACCTGGTCC–3′; HDA C1, sense 5′–ATGAAGCCTCACCGA ATCCGAATG–3′ and antisense 5′–CTTGGTCATCTCCTCAGCGTTGG–3′; HDAC2, sense 5′–CGAGCATCAGACAAGCGGATAGC–3′ and antisense 5′–AGCGACATTCCTACGACCTCCTTC–3′; HDAC3, sense 5′–AACCATGCACCC AGTGTCCA–3′ and antisense 5′–TCTCTTCAGCATCGGCCTCG–3′; HD AC8, sense 5′–TGACTGCCCAGCCACAGAAGG–3′ and antisense 5′–ATGATGCCAC CCTCCAGACCAG–3′; ALD H1A1, sense 5′–ATGTTGACAAAGCTGTGAAGGC–3′ and antisense 5′–ACAAGTACGCATTGGCAAAGAC–3′; ALDH1A2, sense 5′–AACTCAGACTTCGGGCTTGTAG–3′ and antisense 5′–TACTCCCGTAAGCCAA ACTC AC–3′; and ALDH1A3, sense 5′–ATATGTGAGGTGG AAGAAGGCG–3′ and antisense 5′–CCATGGTCTCTAG AGTTGCCAG–3′.

### Protein Extraction and Western Blotting

Total protein was extracted from PFC tissue using radioimmunoprecipitation assay lysis buffer (KeyGEN, China) containing a 0.1% protease inhibitor cocktail (KeyGEN), and protein concentrations were determined using a BCA protein assay kit (ATGene, China) and a microtiter plate reader (Thermo Fisher Scientific, United States). Nucleoproteins were extracted using the Total Histone Extraction Kit (Epigentek, United States) containing a 0.1% protease inhibitor cocktail, and nucleoprotein concentrations were determined using the Detergent Compatible Bradford Protein Assay Kit (Beyotime, China). Western blotting was performed as previously described (Hou et al., 2015). Briefly, proteins were separated using sodium dodecyl sulfate-polyacrylamide gel electrophoresis (SDS-PAGE) and transferred onto 0.20-μm polyvinylidene difluoride (PVDF) membranes (Millipore, United States). All membranes were blocked with 5% bovine serum albumin (BSA) (Solarbio, China) in TBST at room temperature for 1 h. The membranes were incubated with primary antibodies at 4°C overnight and secondary antibodies for 1.5 h at room temperature. An enhanced chemiluminescence (ECL) solution (Millipore, United States) was used to detect the immunocomplexes, and the intensity of the bands was analyzed using ImageJ software (National Institutes of Health, Bethesda, Maryland). Primary antibodies recognizing the following proteins were used for Western blotting (see Supplementary Table 1):

GluN1 (1:1000, ab17345, Abcam, RRID: AB_776808), GluN2A (1:1000, 513863, ZENBIO, RRID: AB_2889880), GluN2B (1:1000, ab183942, Abcam, RRID: AB_2889878), GluA1 (1:1000, ab31232, Abcam, RRID: AB_2113447), GluA2 (1:1000, ab133477, Abcam, RRID: AB_2620181), synapsin 1 (SYN1) (1:1000, ab64581, Abcam, RRID: AB_1281135), AcH3 (1:1000, 06599, Sigma, RRID: AB_2115283), Histone 3 (H3) (1:1000, CY6587, Abways, RRID: AB_2889879), RARα (1:1000, GTX54703, Genetex, RRID: AB_2887874), ALDH1A1 (1:1000, A0157, Abclonal, RRID: AB_2861455), HDAC1 (1:1000, ET1605-35, HuaBio, RRID: AB_2889882), HDAC2 (1:1000, ET1607-78, HuaBio, RRID: AB_2756440), HDAC3 (1:1000, ET1610-5, HuaBio, RRID: AB_2889883), HDAC8 (1:1000, ET1612-90, HuaBio, RRID: AB_2889884), GAPDH (1:5000, HRP60004, Proteintech, RRID: AB_2737588) and PCNA (1:1000, 200947-6B12, ZENBIO, RRID: AB_2722717).

### Chromatin Immunoprecipitation (ChIP) and qPCR (ChIP-qPCR)

ChIP was performed using a ChIP kit (Millipore, United States), and PFC tissue samples were prepared as previously described (Hou et al., 2015). Briefly, fresh pretreated PFC tissue was homogenized, and the chromatin was sheared into 200- to 1000-bp fragments by 40 min of medium-power sonication. The chromatin fragments were incubated with 4 μg of an anti-AcH3 primary antibody (06599, Sigma) or a negative control IgG antibody overnight at 4°C. The specific steps were performed according to the manufacturer’s protocols. qPCR was performed using the SYBR-Green Real-time PCR Kit and a CFX96 real-time PCR detection system, as described above. ChIP signals were quantified as fold enrichment using the comparative ΔΔCt method. A total of seven pairs of primers for sites 0–1000 bp upstream of the rat ALDH1A1 promoter were designed. Only the products amplified with the seventh pair of primers showed significant differences between the CON and VPA groups (data not shown). Therefore, purified DNA was subjected to qPCR with the seventh pair of primers for the ALDH1A1 promoter [sense: 5′–CCCCCTAGCTAAGTCCGA–3′, –1035 to –1018 bp relative to the transcription start site (TSS); antisense: 5′–CTGTGTTGATGCTCCTGC–3′, –919 to –902 bp relative to the TSS].

### Quantitation of RA Using Ultrahigh-Performance Liquid Chromatography/Tandem Mass Spectrometry (UPLC-MS/MS)

As previously reported, all-*trans*-RA levels in the PFC were quantitated using a UPLC-MS/MS method (Xu et al., 2018). Briefly, fresh PFC tissue samples were homogenized with 600 μl of precooled methanol and 20 μl of an internal standard (all-trans-RA-d6, 100 ng/ml). After centrifugation, the supernatant was transferred to a new centrifuge tube and placed in an extractor for draining. The sample was redissolved with 50% acetonitrile. The identity of the fraction containing all-*trans*-RA was determined using an Agilent 6470 UPLC-MS/MS system equipped with a unit for atmospheric pressure chemical ionization (APCI) in the positive ion mode. The amounts of all-*trans*-RA in each fraction were quantitated with calibration curves generated from the standard amounts of all-*trans*-RA. All procedures were performed in a dark environment.

### ALDH Enzyme Activity Assay

ALDH enzyme activity in PFC tissue was determined using the Micro ALDH Assay Kit (Solarbio, China). After fresh tissue was homogenized, the supernatant was collected for detection of ALDH enzyme activity. One nanomole of NADH produced per minute per milligram of protein was defined as 1 unit of ALDH enzyme activity. The rest of the steps followed the manufacturer’s protocols.

### Electrophysiology

Eight-week-old rats were anesthetized via intraperitoneal sodium pentobarbital injection (50 mg/kg) and then underwent cardiac perfusion with a 4°C oxygenated cutting solution. After decapitation, brain tissue was removed from the cranial cavity and transferred to the 4°C oxygenated cutting solution. Each liter of cutting solution contained 1.25 mM NaH_2_PO_4_, 2 mM pyruvate-Na, 26 mM NaHCO_3_, 3 mM KCl, 0.4 mM vitamin C, 2 mM lactate-Na, 220 mM sucrose and 10 mM D-glucose. The olfactory bulb and cerebellum were excised, and the remaining brain tissue was glued vertically to a specimen base and fixed in a specimen tank. The PFC was sliced into 400-μm-thick coronal brain sections using a vibratome. Before recording, the brain sections were incubated in artificial cerebrospinal fluid (ACSF) at 30°C with 95% O_2_ and 5% CO_2_ for at least 1 h. Each liter of ACSF contained 3 mM KCl, 26 mM NaHCO_3_, 2 mM lactate-Na, 124 mM NaCl, 1.25 mM NaH_2_PO_4_, 0.4 mM vitamin C, 10 mM D-glucose, and 2 mM pyruvate-Na. The stimulation electrode was placed in layer V of the PFC, and the glass recording electrode was placed in layers II-III of the PFC to record the field excitatory postsynaptic potentials (fEPSPs) (Rinaldi et al., 2007; Cui et al., 2011; Xu et al., 2012). The stimulus intensity that elicited 50% of the maximum fEPSP amplitude was used as the test spike intensity, and a recording from a 15-min stable period was used as the baseline. Long-term potentiation (LTP) was induced using high-frequency electrical stimulation (HFS; 100 Hz, 1000 ms) and recorded for 45 min (see Supplementary Figure 6; Cui et al., 2011; Hsu et al., 2019). The LTP magnitude was measured as the average potentiation 41–45 min after the onset of HFS induction (Cui et al., 2011; Hsu et al., 2019). Data collection, analyses and processing were performed using pClamp10 software (Molecular Devices).

### Statistical Analysis

Data were analyzed using SPSS 20.0 software, and figures were generated using GraphPad Prism 5.0 software. The Shapiro–Wilk test was used to test normality, and Levene’s test was used to test the homogeneity of variance. Continuous variables with a normal distribution are presented as the mean ± SEM. They were compared using a two-tailed Student’s *t*-test (for two-group comparisons) or one-way ANOVA followed by the *post hoc* Bonferroni test (for multiple-group comparisons). Data with a non-normal distribution are shown as the median (P25–P75) in box plots (center line, median; box limits, upper and lower quartiles; whiskers, minimum and maximum values). Non-normal data were analyzed using non-parametric tests (the Mann–Whitney *U* test for two-group comparisons and the Kruskal–Wallis *H* test followed by the *post hoc* Nemenyi test for multiple-group comparisons). To eliminate the infiuence of sex differences on the results (Kataoka et al., 2013; Beggiato et al., 2017), the offspring used for all experiments in this study were males. Each experiment was repeated at least three times. Statistical significance was set at *P* < 0.05. Based on the study sample size, the effect size and statistical power (1-β err prob) of this study were calculated using *post hoc* analysis with GPower 3.1 software (Ma et al., 2018).

## Results

### Gestational Exposure to VPA Caused Autism-Like Behavioral Deficits and HSP Dysregulation in the PFC in Offspring

**FIGURE 2:**
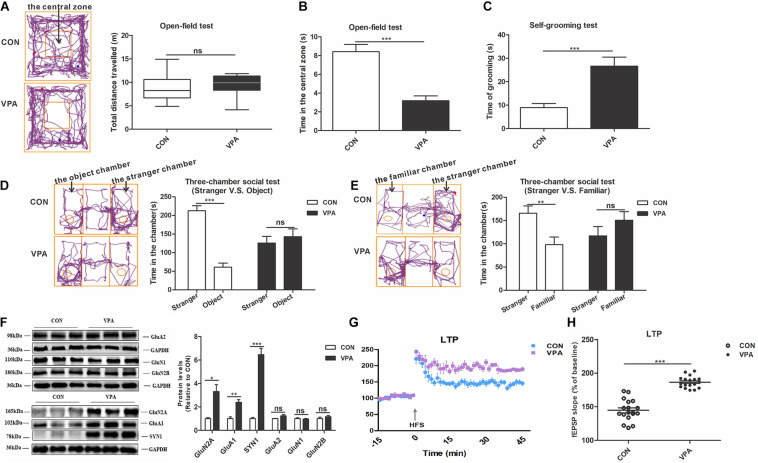
Autism-like behavioral deficits and dysregulation of homeostatic synaptic plasticity in the PFC of VPA-exposed offspring. **(A)** The tracing and total distance traveled in the open-field test (*n* = 20–22 per group, *Z*_1_ = –1.785, *P* = 0.074). **(B)** The time spent in the central zone in the open-field test (*n* = 20–22 per group, *t*_33_ = 5.716, *P* < 0.001). **(C)** The time spent self-grooming in the open-field test (*n* = 20–22 per group, *t*_27_ = –4.131, *P* < 0.001). **(D)** The tracing and social interaction in the three-chamber test (stimulus: a stranger rat vs. an object) (*n* = 20–22 per group, *t*_38_ = 8.692, *P* < 0.001 for CON group; *t*_38_ = –0.61, *P* = 0.546 for VPA group). **(E)** The tracing and recognition of social novelty in the three-chamber test (stimulus: a stranger rat vs. a familiar rat) (*n* = 20–22 per group, *t*_38_ = 2.98, *P* = 0.005 for CON group; *t*_38_ = –1.227, *P* = 0.227 for VPA group). **(F)** Western blot and quantification analyses of GluN2A, GluA1, SYN1, GluA2, GluN1, and GluN2B protein in the PFC of offspring from the CON and VPA groups (*n* = 3 per group). **(G)** Summary graphs of PFC LTP in CON and VPA (*n* = 9 slices from 4 rats per group). **(H)** LTP magnitude was measured as an average potentiation at 41–45 min after the onset of HFS induction (*n* = 9 slices from 4 rats per group). Each experiment was repeated at least three times. The values are the median (P25–P75) **(A)** or means ± SEMs **(B–H)**. Mann–Whitney *U* test **(A)**, Student’s *t*-test **(B–H)**, *P < 0.05, **P < 0.01, ***P < 0.001, ns, not significant; VPA, valproic acid; PFC: prefrontal cortex; PND, Postnatal Day; LTP, long-term potentiation; fEPSP, field excitatory postsynaptic potentials; HFS, high frequency stimulation.

To verify whether prenatal exposure to VPA induces autism-like behaviors, we conducted behavioral tests on offspring, including the open-field and three-chamber social tests (Foley et al., 2012). In the open-field test, there was no noticeable difference in the total distance traveled between the CON and VPA groups, suggesting that VPA exposure does not impair offspring locomotor activity (Figure 2A). VPA offspring spent significantly less time in the central zone and more time on self-grooming than CON offspring (Figures 2B,C), indicating that VPA exposure increases repetitive behaviors and reduces the ability to explore novel environments. The three-chamber social test using a strange rat and an object as stimuli is called the social interaction test (Deacon, 2006; Xu et al., 2018). The other three-chamber social test using a strange rat and a familiar rat as stimuli is called the social novelty test (Deacon, 2006; Xu et al., 2018). In the social interaction test, CON offspring spent significantly more time in the stranger zone than the object zone, but VPA offspring spent almost equal time between the two zones (Figure 2D). In the social novelty test, CON offspring showed more interest in the stranger rat than in the familiar rat, while VPA offspring spent equal time with the two rats (Figure 2E). These results indicate that VPA exposure impairs social interaction and social novelty recognition in offspring. These data suggest that prenatal exposure to VPA replicates the core symptoms of autism.

To assess the impact of prenatal exposure to VPA on HSP in offspring, we investigated the expression levels of proteins related to synaptic plasticity, including AMPA receptors (GluA1 and GluA2), NMDA receptors (GluN1, GluN2A and GluN2B) and SYN1, and the level of long-term potentiation (LTP) in the PFC (Zoghbi, 2003; Grant, 2012; Zoghbi and Bear, 2012). Compared to CON offspring, the VPA offspring showed significantly increased GluN2A, GluA1 and SYN1 protein expression levels in the PFC (Figure 2F). However, the levels of GluA2, GluN1, and GluN2B proteins did not change between the VPA and the CON groups (Figure 2F). The electrophysiological experiment performed in the PFC revealed larger LTP in VPA offspring than in CON offspring (Figures 2G,H). These data indicate that VPA exposure may induce HSP dysregulation by regulating the expression of GluA1, GluN2A and SYN1 in offspring.

### Gestational Exposure to VPA Increased HDAC3 Levels and Downregulated Histone Acetylation Levels in the PFC of Offspring

**FIGURE 3:**
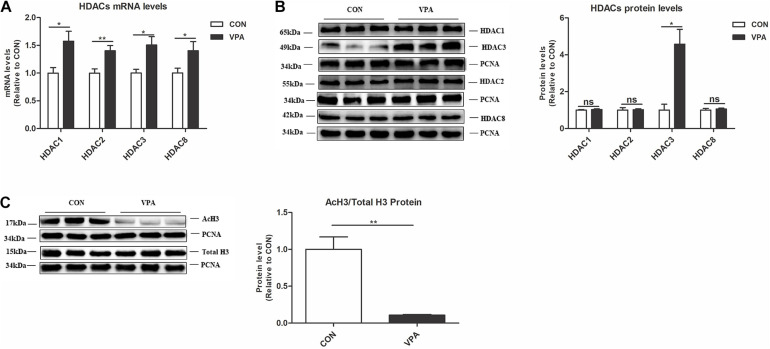
Levels of HDAC3 and histone acetylation in the PFC of VPA-exposed offspring. **(A)** Levels of HDAC1, HDAC2, HDAC3, and HDAC8 mRNA expression in the PFC from the CON and VPA groups, as detected using qPCR and normalized to GAPDH (*n* = 10 per group). **(B)** Western blot and quantification analyses of HDAC1, HDAC2, HDAC3, and HDAC8 protein in the nuclear fraction of PFC from CON and VPA groups (*n* = 3 per group). **(C)** Western blot and quantification analyses of AcH3 and total H3 proteins in the nuclear fraction of PFC from the CON and VPA groups, as normalized to PCNA; the level of histone acetylation was measured as the ratio of AcH3 to H3 (*n* = 3 per group). Each experiment was repeated at least three times. The values are means ± SEMs. Student’s *t*-test, *P < 0.05, **P < 0.01, ns, not significant; VPA, valproic acid; PFC: prefrontal cortex.

To identify potential epigenetic aberrations in the VPA-induced autism model, we examined the levels of class I HDACs (HDAC1, HDAC2, HDAC3, and HDAC8) and AcH3 in the PFC nuclear fraction. HDACs are critical enzymes that function in the epigenetic process by deacetylating histones (Ma et al., 2018). The VPA offspring showed significantly higher HDAC1, HDAC2, HDAC3, and HDAC8 mRNA levels and a higher HDAC3 protein level than the CON offspring (Figures 3A,B). The ratio of AcH3 to H3 in the PFC of VPA offspring was significantly reduced compared to that of CON offspring, indicating the reduction of acetylation level in the VPA offspring (Figure 3C). These data suggest that VPA exposure induces epigenetic abnormalities in histone acetylation in offspring.

### Gestational Exposure to VPA Impaired the ALDH1A1-RA-RARα Pathway in the PFC of Offspring

**FIGURE 4:**
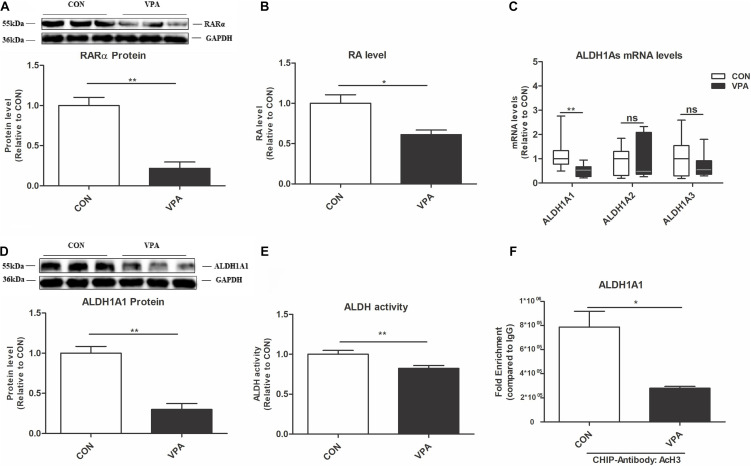
Changes in ALDH1A1 and RA-RARα in the PFC of VPA-exposed offspring. **(A)** Western blot and quantification analyses of RARα protein in the PFC from the CON and VPA groups (*n* = 3 per group). **(B)** RA levels in the PFC from CON and VPA groups, as quantitated using UPLC-MS/MS (*n* = 5 per group). **(C)** ALDH1A1, ALDH1A2, and ALDH1A3 mRNA expression levels in the PFC from the CON and VPA groups, as detected using qPCR and normalized to GAPDH (*n* = 10 per group). **(D)** Western blot and quantification analyses of ALDH1A1 protein in the PFC of offspring from the CON and VPA groups (*n* = 3 per group). **(E)** ALDH enzyme activity in PFC tissue from the CON and VPA groups was determined using an ALDH Assay Kit (*n* = 10 per group). **(F)** ChIP-qPCR analyses of the enrichment of AcH3 on the promoter region of the ALDH1A1 gene in the PFC from the CON and VPA groups (*n* = 3 per group). Each experiment was repeated at least three times. The values are the means ± SEMs **(A,B,D–F)** or median (P25–P75) **(C)**. Student’s *t*-test **(A,B,D–F)**, Mann–Whitney *U* test **(C)**, *P < 0.05, **P < 0.01, ns, not significant; VPA, valproic acid; PFC: prefrontal cortex.

To investigate the possible molecular mechanisms, we evaluated changes in the ALDH1A1-RA-RARα pathway in the PFC of VPA-exposed offspring. The RA-RARα pathway affects HSP and plays a vital role in neurodevelopmental diseases (Aoto et al., 2008; Chen and Napoli, 2008; Zhang et al., 2011; Chen et al., 2014). ALDH1As (ALDH1A1, ALDH1A2, and ALDH1A3) are the key rate-limiting enzymes for RA synthesis (Kumar and Duester, 2011). Compared to those of CON offspring, the levels of RARα protein and RA, the levels of ALDH1A1 mRNA and protein, and the ALDH activity were significantly reduced in the PFC of VPA offspring (Figures 4A–E).

To further investigate the interaction between AcH3 and the ALDH1A1 gene, we conducted a ChIP-qPCR experiment. AcH3 was enriched on the ALDH1A1 gene promoter in the PFC, and this enrichment was significantly decreased in the VPA group compared to the CON group (Figure 4F). These data indicated that the ALDH1A1-RA-RARα pathway is impaired in the VPA-induced autism model, which might be associated with reduced AcH3.

### Treatment With the HDAC Inhibitor MS-275 Rescued the AcH3 Level and the ALDH1A1-RA-RARα Pathway in the PFC of VPA-Exposed Offspring

**FIGURE 5:**
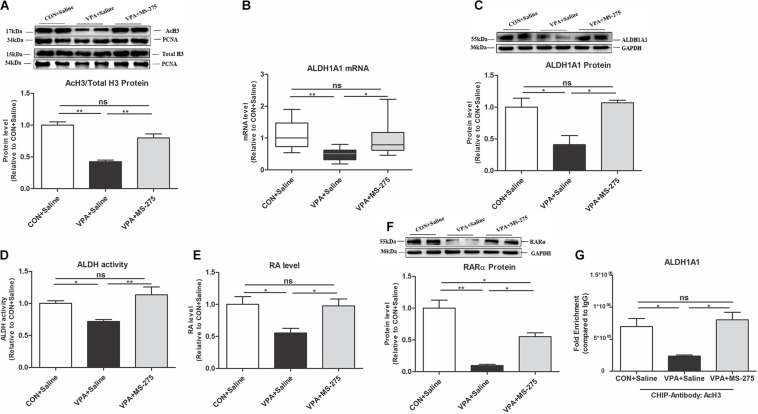
Levels of AcH3, ALDH1A1, and RA-RARα expression in the PFC of VPA-exposed offspring following MS-275 treatment. **(A)** Western blot and quantification analyses of AcH3 and total H3 protein in the nuclear fraction of PFC from the CON + Saline, VPA + Saline, and VPA + MS-275 groups, as normalized to PCNA; the level of histone acetylation was measured as the ratio of AcH3 to H3 (*n* = 3 per group). **(B)** ALDH1A1 mRNA expression in the PFC from the CON + Saline, VPA + Saline, and VPA + MS-275 groups, as detected using qPCR and normalized to GAPDH (*n* = 10 per group). **(C)** Western blot and quantification analyses of ALDH1A1 protein in the PFC of offspring from the CON + Saline, VPA + Saline, and VPA + MS-275 groups (*n* = 3 per group). **(D)** ALDH enzyme activity in PFC tissue from the CON + Saline, VPA + Saline, and VPA + MS-275 groups was determined using the ALDH Assay Kit (*n* = 10 per group). **(E)** RA levels in the PFC from the CON + Saline, VPA + Saline, and VPA + MS-275 groups, as quantitated using UPLC-MS/MS (*n* = 5 per group). **(F)** Western blot and quantification analyses of RARα protein in the PFC from the CON + Saline, VPA + Saline, and VPA + MS-275 groups (*n* = 3 per group). **(G)** ChIP-qPCR analyses of the enrichment of AcH3 on the promoter region of the ALDH1A1 gene in the PFC from the CON + Saline, VPA + Saline, and VPA + MS-275 groups (*n* = 3 per group). Each experiment was repeated at least three times. The values are the means ± SEMs **(A,C–G)** or median (P25–P75) **(B)**. One-way ANOVA with Bonferroni *post hoc* test **(A,C–G)**, Kruskal–Wallis *H* test with Nemenyi *post hoc* test **(B)**, *P < 0.05, **P < 0.01, ns, not significant; VPA, valproic acid; PFC: prefrontal cortex.

To prove that upregulation of the histone acetylation level rescues the impaired ALDH1A1-RA-RARα pathway, we designed an MS-275 treatment experiment. MS-275 is a brain-region-selective class I HDAC inhibitor that is most potent in the PFC (Ma et al., 2018). Systemic administration of MS-275 significantly restored the level of AcH3 in the PFC of VPA-exposed offspring (Figure 5A). After MS-275 treatment in the VPA offspring, the ALDH1A1 mRNA and protein, ALDH activity, RA, and RARα protein levels in the PFC were also significantly elevated (Figures 5B–F). These data suggest that MS-275 treatment rescues the AcH3 level and the ALDH1A1-RA-RARα pathway in the PFC of VPA-exposed offspring.

ChIP-qPCR further showed that MS-275 treatment of VPA-exposed offspring significantly increased the binding of AcH3 to the ALDH1A1 promoter (Figure 5G). This result indicates that AcH3 upregulated the ALDH1A1 expression level via transcription.

### Treatment With the HDAC Inhibitor MS-275 Reversed HSP Deficits and Autism-Like Behavior in VPA-Exposed Offspring

**FIGURE 6:**
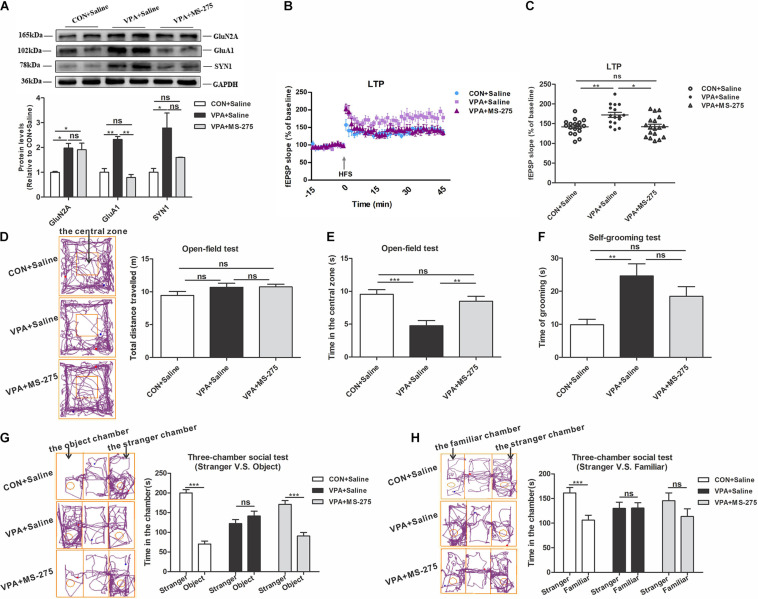
Changes in homeostatic synaptic plasticity and autism-like behaviors in VPA-exposed offspring after MS-275 treatment. **(A)** Western blot and quantification analyses of GluN2A, GluA1, and SYN1 protein in the PFC of offspring from the CON + Saline, VPA + Saline, and VPA + MS-275 groups (*n* = 3 per group). **(B)** Summary graphs of PFC LTP in the CON + Saline, VPA + Saline, and VPA + MS-275 groups (*n* = 9 slices from 4 rats per group). **(C)** LTP magnitude was measured as an average potentiation at 41–45 min after the onset of HFS induction (*n* = 9 slices from four rats per group). **(D–H)** Behavioral tests were performed in offspring from the CON + Saline, VPA + Saline, and VPA + MS-275 groups beginning at PND 43. **(D)** The tracing and total distance traveled in the open-field test (*n* = 20–22 per group, *F*_2,60_ = 1.788, *P* = 0.176). **(E)** The time spent in the central zone in the open-field test (*n* = 20–22 per group, *F*_2,60_ = 11.238, *P* < 0.001). **(F)** The time spent self-grooming in the open-field test (*n* = 20–22 per group, *F*_2,60_ = 7.083, *P* = 0.002). **(G)** The tracing and social interaction in the three-chamber test (stimulus: a stranger rat vs. an object) (*n* = 20-22 per group, *t*_42_ = 11.485, *P* < 0.001 for CON + Saline group; *t*_38_ = –1.199, *P* = 0.238 for VPA + Saline group; *t*_38_ = 6.157, *P* < 0.001 for VPA + MS-275 group). **(H)** The tracing and recognition of social novelty in the three-chamber test (stimulus: a stranger rat vs. a familiar rat) (*n* = 20–22 per group, *t*_42_ = 3.776, *P* < 0.001 for CON + Saline group; *t*_40_ = –0.035, *P* = 0.972 for VPA + Saline group; *t*_42_ = 1.448, *P* = 0.155 for VPA + MS-275 group). Each experiment was repeated at least three times. The values are means ± SEMs. One-way ANOVA with Bonferroni *post hoc* test **(A–F)**, Student’s *t*-test **(G,H)**, *P < 0.05, **P < 0.01, ***P < 0.001, ns, not significant; VPA, valproic acid; PFC, prefrontal cortex; LTP, long-term potentiation; fEPSP, field excitatory postsynaptic potentials; HFS, high frequency train stimulation; PND, Postnatal Day.

Next, we evaluated the improvement of MS-275 on HSP and autism-like behaviors in VPA offspring. MS-275 administration significantly rescued the abnormal GluA1 level in the PFC of VPA-exposed offspring, and the GluN2A and SYN1 levels did not change (Figure 6A). The electrophysiological experiment showed that MS-275 treatment significantly reduced the abnormally high LTP in VPA-exposed offspring (Figures 6B,C). These data indicate that MS-275 treatment improves the dysregulation of HSP in the PFC of VPA-exposed offspring.

In the open-field test, MS-275 treatment of VPA offspring significantly increased the central zone’s time and did not affect the total distance traveled and self-grooming time (Figures 6D–F). In the social interaction test, VPA-exposed offspring treated with MS-275 exhibited a significantly stronger preference for the stranger rat over the object (Figure 6G). In the social novelty test, VPA offspring treated with MS-275 spent equal time with the stranger and familiar rats (Figure 6H). These results suggest that MS-275 partially rescues autism-like behavioral deficits of VPA offspring, such as the abilities to explore novel objects and participate in social interactions.

### Upregulation of RA-RARα Pathway Restored HSP Dysregulation and Autism-Like Behavioral Deficits in VPA-Exposed Offspring

**FIGURE 7:**
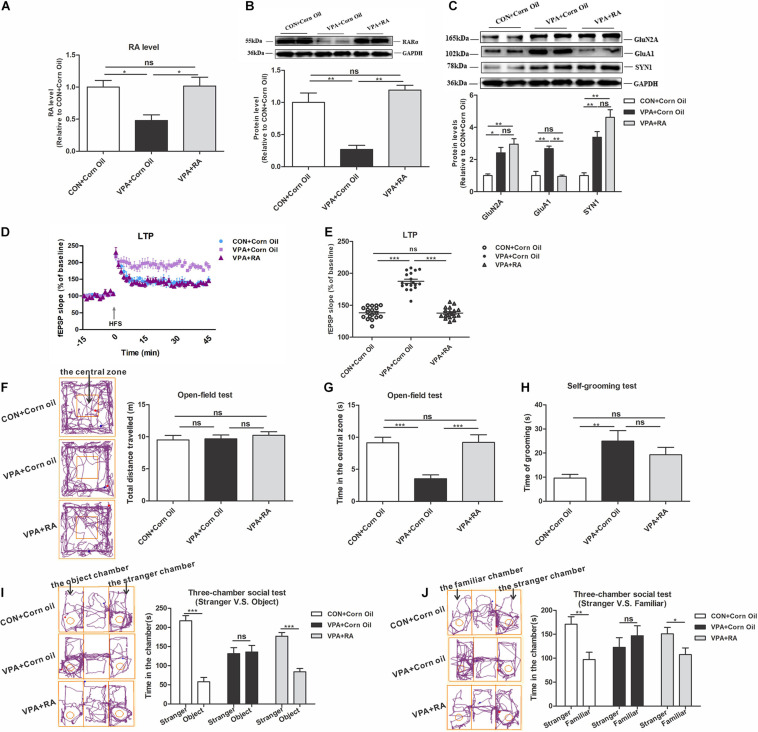
Changes in RARα expression levels, homeostatic synaptic plasticity and autism-like behaviors in VPA-exposed offspring following RA intervention. **(A)** RA levels in the PFC from the CON + Corn Oil, VPA + Corn Oil, and VPA + RA groups, as quantitated using UPLC-MS/MS (*n* = 5 per group). **(B)** Western blot and quantification analyses of RARα protein in the PFC from the CON + Corn Oil, VPA + Corn Oil, and VPA + RA groups (*n* = 3 per group). **(C)** Western blot and quantification analyses of GluN2A, GluA1, and SYN1 protein in the PFC of offspring from the CON + Corn Oil, VPA + Corn Oil, and VPA + RA groups (*n* = 3 per group). **(D)** Summary graphs of PFC LTP in the CON + Corn Oil, VPA + Corn Oil, and VPA + RA groups (*n* = 9 slices from four rats per group). **(E)** LTP magnitude was measured as an average potentiation at 41–45 min after the onset of HFS induction (*n* = 9 slices from four rats per group). **(F–J)** Behavioral tests were performed in offspring from the CON + Corn Oil, VPA + Corn Oil, and VPA + RA groups beginning at PND 43. **(F)** The tracing and total distance traveled in the open-field test (*n* = 20–22 per group, *F*_2,57_ = 0.356, *P* = 0.702). **(G)** The time spent in the central zone in the open-field test (*n* = 20–22 per group, *F*_2,57_ = 12.511, *P* < 0.001). **(H)** The time spent self-grooming in the open-field test (*n* = 20–22 per group, *F*_2,58_ = 5.709, *P* = 0.005). **(I)** The tracing and social interaction in the three-chamber test (stimulus: a stranger rat vs. an object) (*n* = 20–22 per group, *t*_38_ = 9.079, *P* < 0.001 for CON + Corn Oil group; *t*_38_ = –0.191, *P* = 0.85 for VPA + Corn Oil group; *t*_42_ = 7.214, *P* < 0.001 for VPA + RA group). **(J)** The tracing and recognition of social novelty in the three-chamber test (stimulus: a stranger rat vs. a familiar rat) (*n* = 20–22 per group, *t*_38_ = 3.366, *P* = 0.002 for CON + Corn Oil group; *t*_38_ = –0.836, *P* = 0.408 for VPA + Corn Oil group; *t*_40_ = 2.243, *P* = 0.031 for VPA + RA group). Each experiment was repeated at least three times. The values are means ± SEMs. One-way ANOVA with Bonferroni *post hoc* test **(A–H)**, Student’s *t*-test **(I,J)**, *P < 0.05, **P < 0.01, ***P < 0.001, ns = not significant; VPA, valproic acid; PFC, prefrontal cortex; RA, retinoic acid; LTP, long-term potentiation; fEPSP, field excitatory postsynaptic potentials; HFS, high frequency train stimulation; PND, postnatal day.

To further study the causative role of the RA-RARα pathway in the VPA-induced autism model, we designed an RA treatment experiment. Administration of RA significantly enhanced RA and RARα levels in the PFC of VPA offspring (Figures 7A,B). The ALDH1A1, AcH3, and HDAC3 levels were not significantly changed after RA treatment (data not shown).

We then explored the effect of RA treatment on the HSP and autism-like behavior in VPA-exposed offspring. The level of GluA1 was significantly reduced in VPA offspring treated with RA (Figure 7C). The Electrophysiological experiment in the PFC showed that RA treatment reduced the LTP level in VPA-exposed offspring (Figures 7D,E). These data indicate that the recovery of RA-RARα pathway rescues impaired HSP in the PFC of VPA-exposed offspring. In the open-field test, RA treatment of VPA offspring significantly increased the central zone’s time and did not change the total distance traveled and self-grooming time (Figures 7F–H). In the three-chamber social test, VPA offspring treated with RA spent more time with the stranger rat over the object and showed a stronger preference for the stranger rat over the familiar rat (Figures 7I,J). These results suggest that RA partially restores autism-like behavioral deficits in VPA offspring, such as the abilities to explore novel things, participate in social interaction and recognize social novelty.

## Discussion

Prenatal VPA exposure is an apparent risk factor for ASD, and the VPA-induced autism model is a classic model for studying the neurobiology underlying ASD (Mabunga et al., 2015). Our study showed that prenatal exposure to VPA replicates the core symptoms of autism, suggesting that the VPA-induced autism model was successfully constructed. Recent studies have validated HSP dysregulation as a major hallmark of ASD (Bagni and Zukin, 2019). AMPA receptors (GluA1 and GluA2), NMDA receptors (GluN1 and GluN2) and SYN1 are critical molecular markers of synaptic plasticity, and LTP is an essential form of synaptic plasticity (Bagni and Zukin, 2019). Our study found the widely upregulated protein levels (GluN2A, GluA1, and SYN1) and enhanced LTP in the PFC of VPA-exposed offspring, consistent with previous reports (Rinaldi et al., 2007; Markram et al., 2008). These results indicate that HSP dysregulation caused by GluN2A, GluA1, and SYN1 aberrants is a vital pathological feature of the VPA-induced autism model.

Epigenetic modification is important in the heterogeneous pathogenesis of ASD (Ma et al., 2018; Rylaarsdam and Guemez-Gamboa, 2019). HDACs perform a major epigenetic process by deacetylating histones (Ma et al., 2018) and play pivotal roles in neurodegenerative diseases, including ASD (Guan et al., 2009; Fischer et al., 2010; Gräff et al., 2012; Autism Spectrum Disorders Working Group of The Psychiatric Genomics Consortium, 2017). VPA inhibits HDAC activity and impairs histone acetylation (Phiel et al., 2001; Kazantsev and Thompson, 2008), which may be responsible for VPA-induced teratogenesis (Phiel et al., 2001), birth defects (Phiel et al., 2001) and even autism-like behaviors. One previous study found that prenatal VPA exposure caused a transient increase in acetylated histone levels in the embryonic brain, which may be due to the direct inhibitory effect of VPA on HDAC activity (Kataoka et al., 2013). However, our study observed that VPA exposure reduced the AcH3 level in the postnatal brain in offspring, consistent with the report by Kazlauskas et al. (2016). It is speculated that prenatal VPA exposure indirectly induces the overexpression of HDACs via negative feedback, leading to an imbalance in epigenetic regulation. The increased HDAC expression in VPA offspring in our study also confirmed this speculation. The reasons for the upregulation of HDAC levels must be further studied. All these findings suggest that dysfunction in acetylation epigenetics may be closely involved in the pathogenesis of VPA-induced autism-like behaviors.

To identify the precise mechanisms of epigenetic modification, we also examined the change in the ALDH1A1-RA-RARα pathway in the PFC of the VPA-induced autism model. The RA-RARα pathway regulates the expression of proteins associated with synaptic plasticity, such as GluA1, and plays a critical causative role in neurological diseases (Aoto et al., 2008; Chen and Napoli, 2008; Chen et al., 2014). ALDH1A family proteins are key rate-limiting enzymes in RA synthesis (Kumar and Duester, 2011; Zhang et al., 2017). Some studies have found that variants in the ALDH1A family genes are clinically related to ASD (Galter et al., 2003; Wan et al., 2009; Fares-Taie et al., 2013). Another report confirmed that administration of an ALDH1A antagonist induced autism-like symptoms in mice (Xu et al., 2018). Our study showed a reduction of ALDH1A1 level and ALDH activity in VPA-exposed offspring, consistent with the decreased RA and RARα protein levels. These results indicate that decreased ALDH1A1-RA-RARα pathway might play a key role in the VPA-induced autism model. Previous studies have reported that histone acetylation levels regulate ALDH1A1 expression levels via transcription in cholangiocarcinoma (Schilderink et al., 2016; Yoshino et al., 2020). Our ChIP-qPCR experiment showed that AcH3 enrichment on the ALDH1A1 gene promoter was decreased in the PFC of VPA offspring. Considering the parallel downregulation of AcH3, AcH3 enrichment and ALDH1A1 levels, we hypothesized that the decreased ALDH1A1 levels may be associated with reduced AcH3.

The causal regulatory relationship between AcH3 and ALDH1A1 was studied. We observed that HDAC inhibitor MS-275 treatment of VPA offspring increased acetylation levels, followed by the upregulation of ALDH1A1 level. The impaired RA-RARα pathway and most autism-like synaptic and behavioral deficits were also significantly rescued. ChIP-qPCR further showed that AcH3 enrichment on the ALDH1A1 gene promoter after MS-275 treatment was elevated, consistent with the upregulation of the ALDH1A1 level. Together, these results clarify that the decreased histone acetylation downregulates ALDH1A1 expression via transcription and impairs the RA-RARα pathway, thereby leading to autism-like synaptic and behavioral deficits in VPA-exposed offspring.

Subsequently, in the RA treatment experiment, we demonstrated that the RA-RARα pathway upregulation reduced GluA1 level and HSP, thereby partially rescuing most autism-like behaviors in VPA-exposed offspring. The ALDH1A1 and AcH3 levels were not changed after RA treatment (data not shown). These results fully prove that the RA-RARα pathway plays a critical causative role downstream of ALDH1A1 in the VPA-induced autism model. Xu et al. (2018) also identified impaired RA homeostasis as a key mechanism underlying the UBE3A hyperactivity-induced autism model. In their autism model, RA reduction decreased the binding to RARα and elevated the binding of RARα to GluA1 mRNA without impairing the RARα level, which reduced GluA1 translation and HSP (Poon and Chen, 2008; Xu et al., 2018). In our study, the increase in HSP might be due to the downregulation of RARα, which reduced the binding of RARα to GluA1 mRNA and thus increased GluA1 translation (Poon and Chen, 2008; Hsu et al., 2019).

Notably, Foley et al. (2012) and our studies both found that HDAC inhibitors improved VPA-induced autism-like behavioral deficits via HSP restoration. HDAC inhibitors also showed significant therapeutic effects on behavioral deficits in the Shank3-deficient autism model via HSP regulation (Ma et al., 2018). These results suggest that HDAC-targeting agents have great potential for the treatment heterogeneous autism due to their extensive regulatory effects on HSP (Ma et al., 2018). This provides new ideas for the treatment of ASD and is worthy of in-depth study.

One limitation of our study was that autism-like synaptic and behavioral deficits were not wholly remedied by MS-275 treatment, suggesting that there may be other pathogenic mechanisms in the VPA-induced autism model.

## Conclusion

Our study first found that VPA caused autism-like synaptic and behavioral deficits by impairing the histone acetylation of ALDH1A1 and thus downregulating the RA-RARα pathway, suggesting a precise epigenetic mechanism underlying the VPA-induced autism model. Synaptic and behavioral deficits were significantly rescued by regulating the histone acetylation of ALDH1A.

## Data Availability Statement

The raw data supporting the conclusions of this article will be made available by the authors, without undue reservation.

## Ethics Statement

The animal study was reviewed and approved by the Animal Experimentation Ethics Committee of Chongqing Medical University (Chongqing, China) and conducted in accordance with the guidelines of the Institutional Animal Care and Use Committee of Chongqing Medical University.

## Author Contributions

HL: methodology, investigation, formal analysis, and writing an original draft. MT: investigation, software, and formal analysis. BC: investigation and software. SW, LX, JZ, QW, XL, and QZ: investigation. JC: conceptualization, methodology, supervision, validation, writing – review and editing. TL: conceptualization, funding acquisition, supervision, validation, writing – review and editing. All authors contributed to the article and approved the submitted version.

## Conflict of Interest

The authors declare that the research was conducted in the absence of any commercial or financial relationships that could be construed as a potential conflict of interest.
