# Supplementary material for: Valproic Acid Induces Autism-Like Synaptic and Behavioral Deficits by Disrupting Histone Acetylation of Prefrontal Cortex ALDH1A1 in Rats
Source: Front Neurosci. 2021 Apr 28;15:641284. doi: 10.3389/fnins.2021.641284 (PMC8113628; doi:10.3389/fnins.2021.641284)
Supplement: Supplementary file 1 [file Table_1.DOCX]

| **Supplementary table 1 RRID of primary antibodies used** | | | |
| --- | --- | --- | --- |
| Name of antibody | Catalog number | Vendor | RRID |
| Anti-GluN1 | ab17345 | Abcam | AB_776808 |
| Anti-GluN2A | 513863 | ZENBIO | AB_2889880 |
| Anti-GluN2B | ab183942 | Abcam | AB_2889878 |
| Anti-GluA1 | ab31232 | Abcam | AB_2113447 |
| Anti-GluA2 | ab133477 | Abcam | AB_2620181 |
| Anti-SYN1 | ab64581 | Abcam | AB_1281135 |
| Anti-AcH3 | 06599 | Sigma | AB_2115283 |
| Anti-H3 | CY6587 | Abways | AB_2889879 |
| Anti-RARα | GTX54703 | Genetex | AB_2887874 |
| Anti-ALDH1A1 | A0157 | Abclonal | AB_2861455 |
| Anti-HDAC1 | ET1605-35 | HuaBio | AB_2889882 |
| Anti-HDAC2 | ET1607-78 | HuaBio | AB_2756440 |
| Anti-HDAC3 | ET1610-5 | HuaBio | AB_2889883 |
| Anti-HDAC8 | ET1612-90 | HuaBio | AB_2889884 |
| Anti-GAPDH | HRP60004 | Proteintech | AB_2737588 |
| Anti-PCNA | 200947-6B12 | ZENBIO | AB_2722717 |
| GluN: NMDA receptor; GluA: AMPA receptor; SYN1: Synapsin1; AcH3: Acetylated histone 3; H3: Histone 3; RARα: Retinoic acid receptor α; ALDH1A1: Acetaldehyde dehydrogenase 1A1; HDAC: Histone deacetylase; GAPDH: Glyceraldehyde-3-phosphate dehydrogenase; PCNA: Proliferating cell nuclear antigen. | | | |
