# Supplementary material for: Valproic Acid Induces Autism-Like Synaptic and Behavioral Deficits by Disrupting Histone Acetylation of Prefrontal Cortex ALDH1A1 in Rats
Source: Front Neurosci. 2021 Apr 28;15:641284. doi: 10.3389/fnins.2021.641284 (PMC8113628; doi:10.3389/fnins.2021.641284)
Supplement: Supplementary file 2 [file Table_2.DOCX]

**Legends**

**Supplementary figure 1 The impacts of MS-275 administrations on behavioral and electrophsiological performances of control rats.** (A) The total distance travelled in the open-ﬁeld test (n=20-22 per group). (B) The time spent in the central zone in the open-ﬁeld test (n=20-22 per group). (C) The time spent in self-grooming in the open-ﬁeld test (n=20-22 per group). (D) Social interaction in the three-chamber test (stimulus: a stranger rat vs. an object) (n=20-22 per group). (E) Recognition of social novelty in the three-chamber test (stimulus: a stranger rat vs. a familiar rat) (n=20-22 per group). (F) Summary graphs of PFC LTP in CON+Saline group and CON+MS-275 group (n=9 slices from 4 rats per group). (G) LTP magnitude was measured as average potentiation at 41-45 min after onset of HFS induction (n=9 slices from 4 rats per group). Each experiment was repeated at least three times. The values are the means ± SEMs. Student’s *t*-test, **p* < 0.05, ***p* < 0.01, ****p* < 0.001, ns = not signiﬁcant; PFC: prefrontal cortex; LTP, long-term potentiation; fEPSP, ﬁeld excitatory postsynaptic potentials; HFS, high frequency train stimulation.

**Supplementary figure 2 The impacts of RA administrations on behavioral and electrophsiological performances of control rats.** (A) The total distance travelled in the open-ﬁeld test (n=20-22 per group). (B) The time spent in the central zone in the open-ﬁeld test (n=20-22 per group). (C) The time spent in self-grooming in the open-ﬁeld test (n=20-22 per group). (D) Social interaction in the three-chamber test (stimulus: a stranger rat vs. an object) (n=20-22 per group). (E) Recognition of social novelty in the three-chamber test (stimulus: a stranger rat vs. a familiar rat) (n=20-22 per group). (F) Summary graphs of PFC LTP in CON+Corn Oil group and CON+RA group (n=9 slices from 4 rats per group). (G) LTP magnitude was measured as average potentiation at 41-45 min after onset of HFS induction (n=9 slices from 4 rats per group). Each experiment was repeated at least three times. The values are the means ± SEMs. Student’s *t*-test, **p* < 0.05, ***p* < 0.01, ****p* < 0.001, ns = not signiﬁcant; RA, Retinoic acid; PFC: prefrontal cortex; LTP, long-term potentiation; fEPSP, ﬁeld excitatory postsynaptic potentials; HFS, high frequency train stimulation.

**Supplementary figure 3 Schematic diagram of experimental design about treatment time.** (A) Schematic diagram of the CON+Saline, VPA+Saline and VPA+MS-275 groups at different treatment time (3 days, 1 week, 2 weeks, 3 weeks or 4 weeks prior to behavioral testing). VPA+MS-275 offspring (n=28-30) received VPA during pregnancy and an intraperitoneal injection of 3.5 mg/kg MS-275 for 3 days, 1 week, 2 weeks, 3 weeks or 4 weeks prior to behavioral testing, respectively. A comparison was made to saline-treated offspring from dams that had received saline (CON+Saline, n=28-30) or VPA (VPA+Saline, n=28-30) during pregnancy. (B) Schematic diagram of the CON+Corn Oil, VPA+Corn Oil and VPA+RA groups at different treatment time (3 days, 1 week, 2 weeks, 3 weeks or 4 weeks prior to behavioral testing). VPA+RA offspring (n=28-30) received VPA during pregnancy and oral administration of 6 mg/kg RA once daily for 3 days, 1 week, 2 weeks, 3 weeks or 4 weeks prior to behavioral testing, respectively. A comparison was made to corn oil-treated offspring from dams that had received saline (CON+Corn Oil, n=28-30) or VPA (VPA+Corn Oil, n=28-30) during pregnancy. Behavioural tests were conducted beginning at PND 43, and the oﬀspring were sacriﬁced for brain tissue collection after behavioural tests. VPA, valproic acid; RA, Retinoic acid; PND, Postnatal Day.

**Supplementary figure 4 Changes in autism-like behaviours in VPA offspring after MS-275 treatment for 3 days, 1 week, 2 weeks, 3 weeks and 4 weeks prior to behavioral testing, respectively.** (A) The total distance travelled in the open-ﬁeld test (n=20-22 per group). (B) The time spent in the central zone in the open-ﬁeld test (n=20-22 per group). (C) The time spent in self-grooming in the open-ﬁeld test (n=20-22 per group). (D) Social interaction in the three-chamber test (stimulus: a stranger rat vs. an object) (n=20-22 per group). (E) Recognition of social novelty in the three-chamber test (stimulus: a stranger rat vs. a familiar rat) (n=20-22 per group). Each experiment was repeated at least three times. The values are the means ± SEMs. One-way ANOVA with Bonferroni post hoc test (A-C), Student’s *t*-test (D, E), **p* < 0.05, ***p* < 0.01, ****p* < 0.001, ns = not signiﬁcant; VPA, valproic acid**.**

**Supplementary figure 5 Changes in autism-like behaviours in VPA offspring after RA treatment for 3 days, 1 week, 2 weeks, 3 weeks and 4 weeks prior to behavioral testing, respectively.** (A) The total distance travelled in the open-ﬁeld test (n=20-22 per group). (B) The time spent in the central zone in the open-ﬁeld test (n=20-22 per group). (C) The time spent in self-grooming in the open-ﬁeld test (n=20-22 per group). (D) Social interaction in the three-chamber test (stimulus: a stranger rat vs. an object) (n=20-22 per group). (E) Recognition of social novelty in the three-chamber test (stimulus: a stranger rat vs. a familiar rat) (n=20-22 per group). Each experiment was repeated at least three times. The values are the means ± SEMs. One-way ANOVA with Bonferroni post hoc test (A-C), Student’s *t*-test (D, E), **p* < 0.05, ***p* < 0.01, ****p* < 0.001, ns = not signiﬁcant; VPA, valproic acid; RA, Retinoic acid.

**Supplementary figure 6** **The LTP at different times after induction.** (A) Summary graphs of LTP during 80 min onset of HFS induction in the PFC of the CON and VPA groups (n=9 slices from 4 rats per group). (B) LTP magnitude was measured as an average potentiation at 41-45 min, 56-60min and 76-80min after onset of HFS induction (n=9 slices from 4 rats per group). Each experiment was repeated at least three times. The values are the means ± SEMs. One-way Repeated Measures ANOVA with Bonferroni post hoc test, **p* < 0.05, ***p* < 0.01, ****p* < 0.001, ns = not signiﬁcant; VPA, valproic acid; PFC: prefrontal cortex; LTP, long-term potentiation; fEPSP, ﬁeld excitatory postsynaptic potentials; HFS, high frequency train stimulation.
