# Supplementary figures and images for: Valproic Acid Induces Autism-Like Synaptic and Behavioral Deficits by Disrupting Histone Acetylation of Prefrontal Cortex ALDH1A1 in Rats
Source: Front Neurosci. 2021 Apr 28;15:641284. doi: 10.3389/fnins.2021.641284 (PMC8113628; doi:10.3389/fnins.2021.641284)

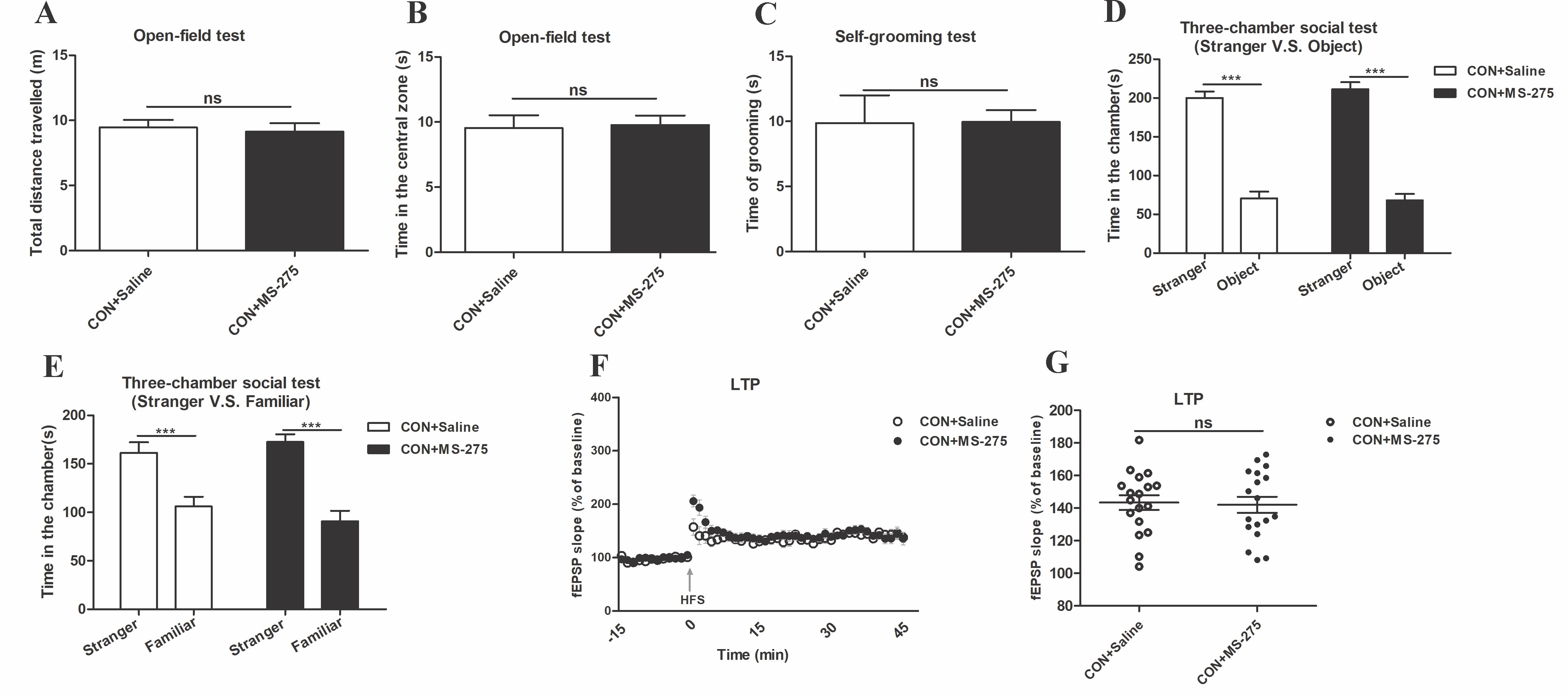

Supplement: Supplementary file 3 [file Image_1.JPEG]

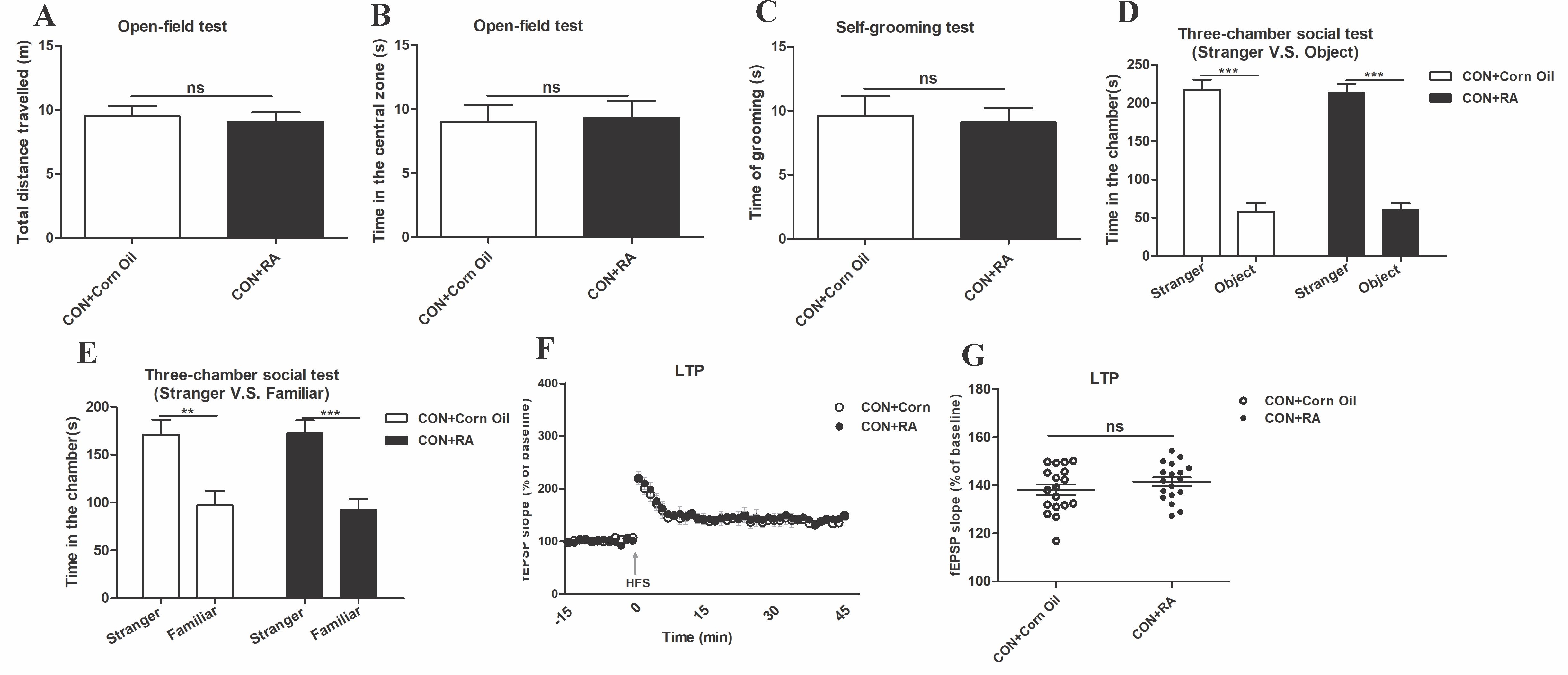

Supplement: Supplementary file 4 [file Image_2.JPEG]

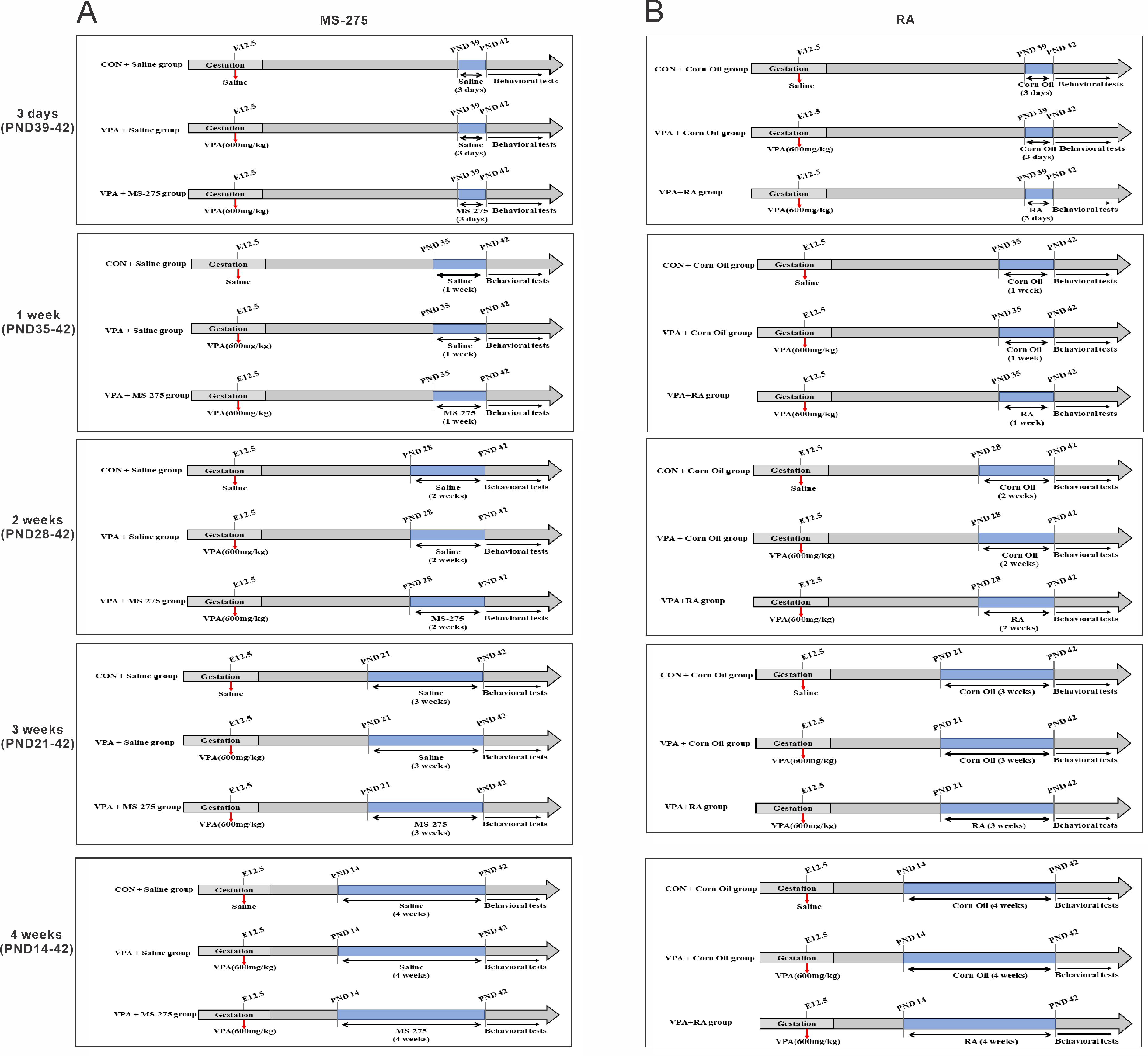

Supplement: Supplementary file 5 [file Image_3.JPEG]

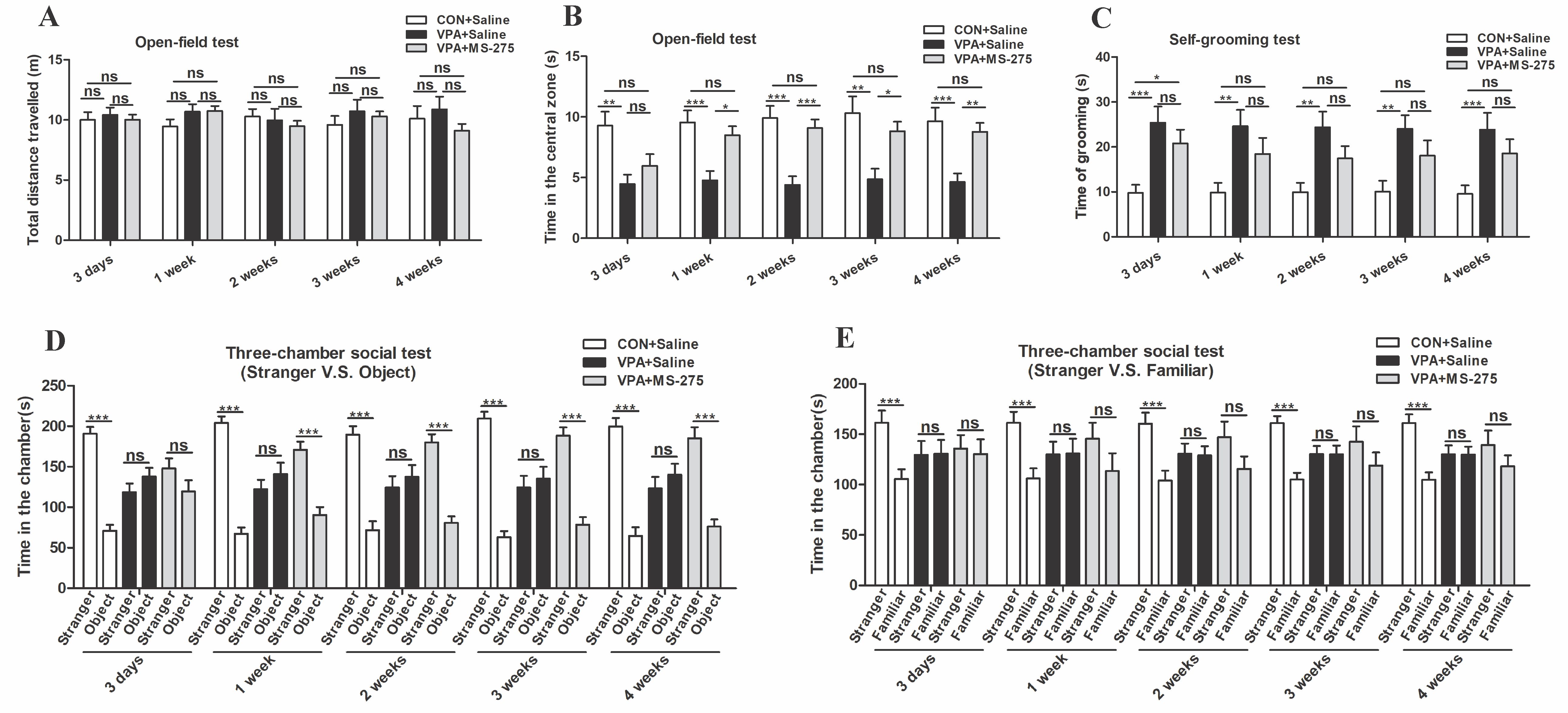

Supplement: Supplementary file 6 [file Image_4.JPEG]

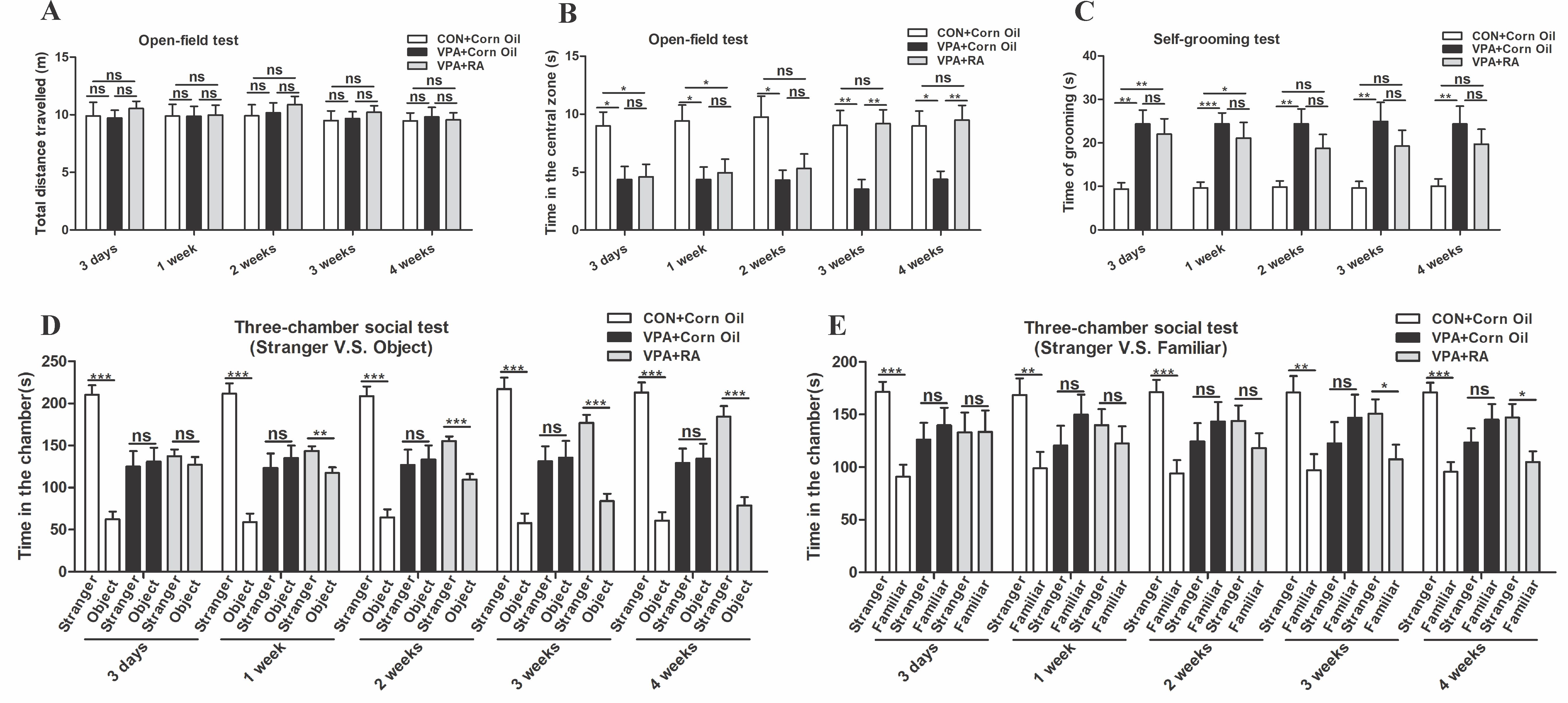

Supplement: Supplementary file 7 [file Image_5.JPEG]

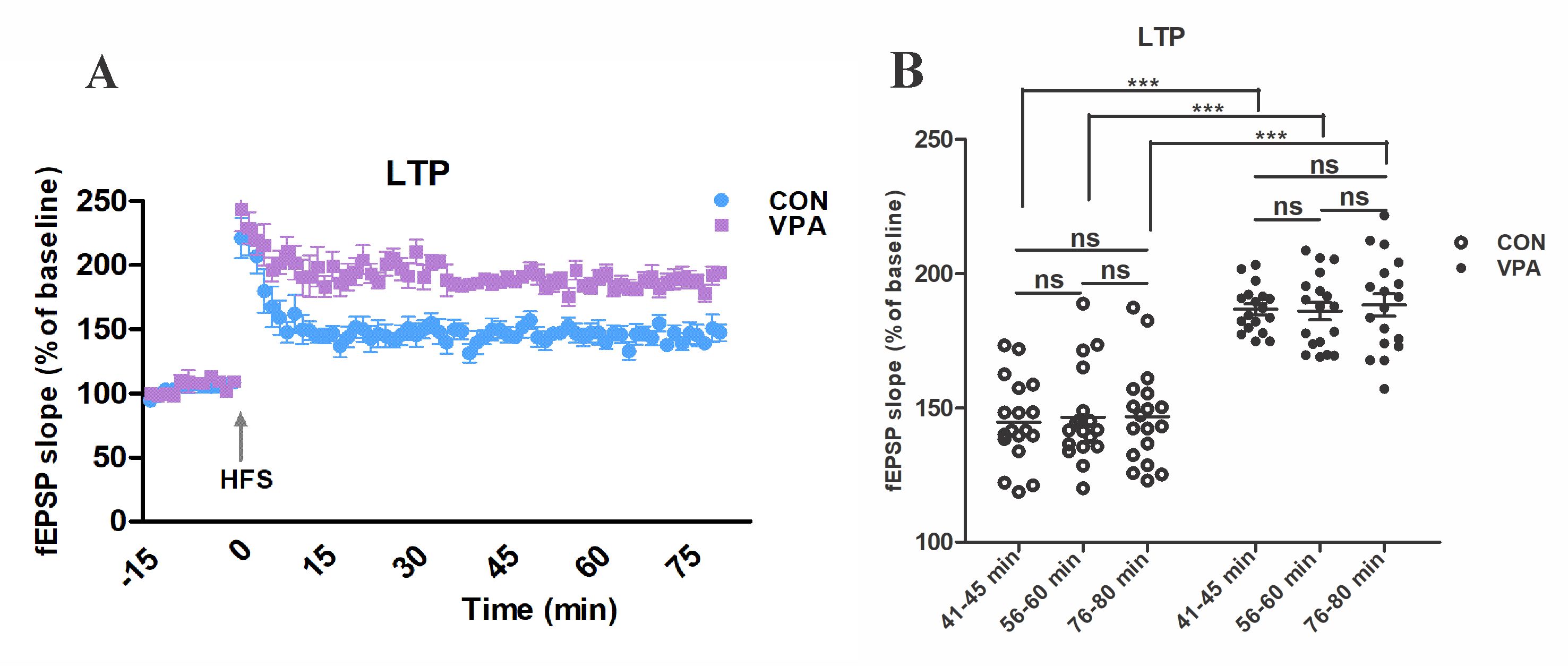

Supplement: Supplementary file 8 [file Image_6.JPEG]
